# Supplementary material for: Analysis of PPARγ Signaling Activity in Psoriasis
Source: Int J Mol Sci. 2021 Aug 10;22(16):8603. doi: 10.3390/ijms22168603 (PMC8395241; doi:10.3390/ijms22168603)
Supplement: Supplementary file 1 [file ijms-22-08603-s001.zip › Supplemental materials_Analysis of PPARg signaling activity in psoriasis/Pathway models/Models images and html files/PPARG expressed in cells assotiated with psoriasis/213437.html]

sebaceous gland


# Organ sebaceous gland

|  |  |
| --- | --- |
| URN | urn:agi-ncimorgan:C0036505 |
| Total Entities | 4 |
| Connectivity | 704 |
| Name | sebaceous gland |

---

|  |  |
| --- | --- |
| ChildConcepts | duct of sebaceous gland |
|  | gland of Zeis |
|  | meibomian gland |
|  | preputial gland |

---

|  |  |
| --- | --- |
| Pathway | PPARG expressed in cells assotiated with psoriasis |

---

|  |  |
| --- | --- |
| MedScan ID | 8803070 |

---

|  |  |
| --- | --- |
| Alias | sebaceous gland |
|  | sebum gland |
|  | sebum glands |
|  | glandulae sebaceae |
|  | glandula sebacea |
|  | sebaceous glands |

---

|  |  |
| --- | --- |
| NCIm ID | C0036505 |

---
